# The outcomes of epiretinal membrane peeling in patients with foveal herniation

**DOI:** 10.1186/s40942-018-0145-8

**Published:** 2018-11-07

**Authors:** Abdullah Ozkaya, Gurkan Erdogan, Gokhan Demir

**Affiliations:** Beyoglu Eye Training and Research Hospital, Bereketzade Cami Sok., 34421 Beyoglu, Istanbul, Turkey

**Keywords:** Epiretinal membrane, Foveal herniation, Vitrectomy

## Abstract

**Background:**

To evaluate the surgical outcomes of vitrectomy in patients with foveal herniation associated with epiretinal membrane (ERM).

**Methods:**

This was a retrospective case series. The patients who had a foveal herniation associated with ERM, underwent vitrectomy, and had a minimum follow-up period of 12 months were included. The visual and anatomical outcomes were assessed during the first 12 months of follow-up and at the last follow-up visit. The main outcome measure was the reorganization pattern of foveal pit at month 12.

**Results:**

Eleven eyes of 11 patients were included. The mean postoperative follow-up period was 14.8 ± 2.6 months. The foveal contour was completely restored in 5 eyes (45.5%), partially restored in 5 eyes (45.5%), and was not restored in 1 eye (9.1%) at postoperative month 12 follow-up visit. The mean preoperative best corrected visual acuity was 0.61 ± 0.16 LogMAR and increased to 0.49 ± 0.16 LogMAR at month 12 (*p* < 0.0001). The mean preoperative central retinal thickness was 476 ± 128 micrometers and decreased to 302 ± 70 micrometers at month 12 (*p* < 0.0001).

**Conclusion:**

The foveal contour was restored in 45.5% of the eyes and visual acuity was significantly increased by a mean of 1.2 LogMAR lines at month 12 in patients with foveal herniation associated with ERM.

## Background

Epiretinal membrane (ERM) is the most frequent vitreomacular surface disorder [1–4]. Surgical treatment is usually required if it causes metamorphopsia and visual deterioration, and consists of vitrectomy and ERM peeling [4–6]. After the introduction of optical coherence tomography (OCT), different ERM subtypes have been described [7–9]. The ERMs have been classified into diffuse, cystoid macular edema, vitreomacular traction, and pseudolamellar hole subtypes [7]. An interesting and new subtype of ERM was described by Ozdemir and Karacorlu in 2017 as ‘foveal herniation’’ [10]. This subtype was previously classified as “outer retinal inward projection and inner retinal thickening” by Hwang et al. [8]. However, there is little data with regards to the surgical outcomes of this specific subtype. Therefore, we aimed to investigate the surgical outcomes of the ERM patients with foveal herniation.

## Methods

The records of the ERM patients who underwent 23-gauge vitrectomy between January 2016 and February 2017 were reviewed for this retrospective case series. A total of 11 patients showed foveal herniation and were included in the study. A written consent was obtained from all of the patients preoperatively. Local review board approval was obtained and the study was in adherence with the tenets of the Declaration of Helsinki.

**Fig. 1:**
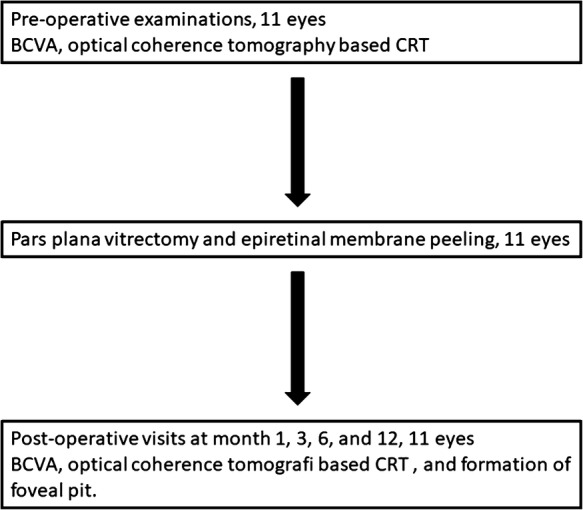
The flow chart of the study method

**Fig. 2:**
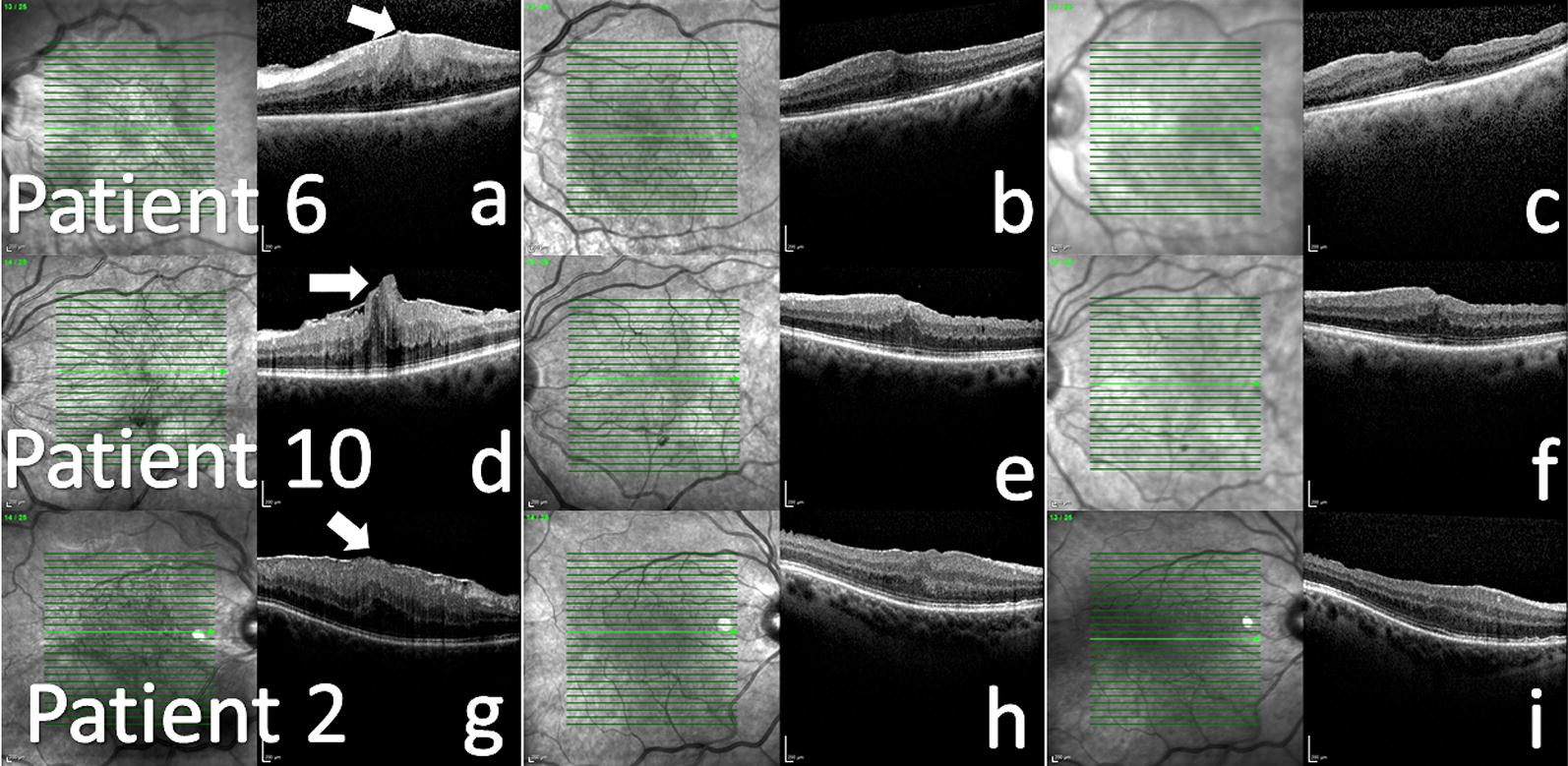
The examples of eyes with fully, partially or not restored foveal contour, **a**–**c** the optical coherence tomography scans of patient 6 at baseline, postoperative month 3 and 12, who showed fully restored foveal pit at month 12. White arrow indicated the tiny foveal herniation at baseline, **d**–**f** the optical coherence tomography scans of patient 10 at baseline, postoperative month 3 and 12, who showed partially restored foveal pit at month 12. White arrow indicated the prominent foveal herniation at baseline, **g**–**i** the optical coherence tomography scans of patient 2 at baseline, postoperative month 3 and 12, in whom foveal pit was not restored at month 12. White arrow indicated the tiny foveal herniation at baseline

The patients with a diagnosis of idiopathic ERM and who showed foveal herniation and had a minimum postoperative follow-up period of 12 months were included (Fig. 1). The patients who had a concomitant vascular retinal disease were not included. Age, gender, best corrected visual acuity (BCVA), central retinal thickness (CRT), and complications were collected retrospectively from the patients’ medical records. The patients underwent a standard ocular examination which included BCVA measurement via a projection chart in decimals, biomicroscopy (Heig Streit AG, Koning Switzerland), intraocular pressure measurement via applanation tonometer (Heig Streit AG, Koning Swizerland), and fundus examination via 90 diopter lens (Volk Optical Inc, Mentor, Ohio, USA). Optical coherence tomography imaging was performed (Spectralis; Heidelberg Engineering, Heidelberg, Germany) preoperatively. The examinations were repeated at post-operative day 1, week 1, and months 1, 3, 6, and 12. Central retinal thickness, defined as the mean thickness of the neurosensory retina in a central 1 mm diameter area, was computed using OCT mapping software generated by the device. The diagnosis of foveal herniation was made as previously described [10]. The patients who showed a protruded retinal tissue through the ERM were diagnosed with foveal herniation (Fig. 2). At postoperative month 12 the pattern of the formation of foveal contour was divided into 3 groups. The foveal pit was accepted as fully restored if there was a well-formed foveal pit on the OCT scan, it was accepted as partially restored if a pit was detected but was not fully formed, and was accepted as not restored if a pit was not detected.

### Surgical technique

All of the operations were performed by two experienced vitreoretinal surgeons (A.O. and G.E.). The patients underwent 23-gauge vitrectomy with the Constellation system (Alcon Surgical, Ft. Worth, TX). A wide-field viewing system was used. Patients with a significant cataract underwent simultaneous phacoemulsification in the same session with the vitrectomy procedure.

**Fig. 3:**
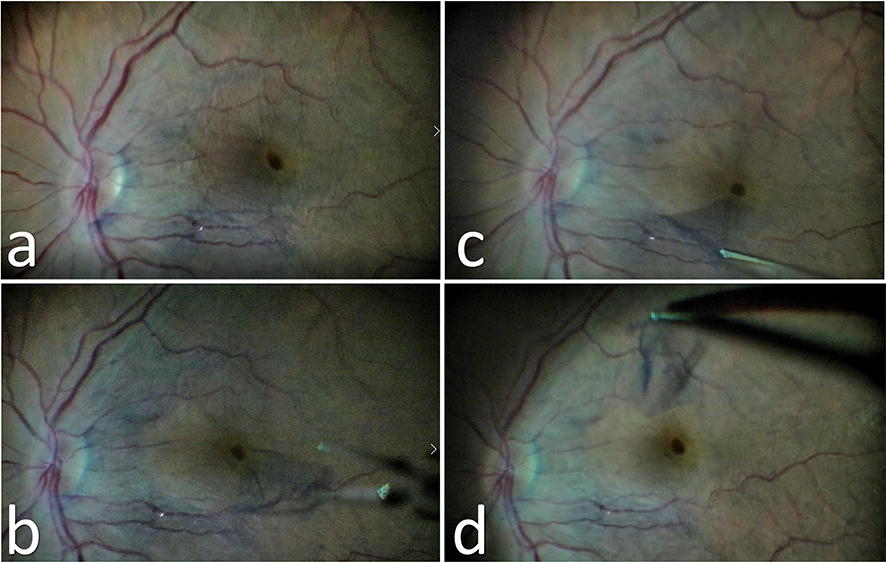
The peroperative screenshots of a patient, **a** the epiretinal membrane was stained with trypan-blue and the foveal herniation is detected like a pseudohole, **b**, **c** epiretinal membrane is being peeled and it is clearly seen that it has an operculum at the center, **d** the end of the peeling foveal herniation is still detected like a pseudohole

After preparing the three standard ports for vitrectomy, core vitrectomy was performed and posterior vitreous was checked for the presence of a posterior vitreous detachment (PVD) with the help of triamcinolone and PVD was induced if was not present. Then a limited posterior vitrectomy was performed as previously described [11]. Epiretinal membrane was stained with trypan-blue (0.06%, VisionBlue, DORC, Netherlands) and peeled via a 23-gauge microforceps. After peeling the ERM, the continuity of the internal limiting membrane (ILM) was checked with the help of brilliant blue G (Fluoron Gmbh, NEU-Ulm, Germany) and ILM was also peeled if it was suspiciously or clearly disrupted. At least two-disc diameters of ERM was peeled (Fig. 3). Retina was examined with indentation at the end of the surgery to detect new or previous retinal breaks and barrier laser photocoagulation was performed when required. Tamponade choice was not based on any specific rules and positioning was not suggested according to the used tamponade.

Primary outcome measure was the formation pattern of foveal contour at month 12 after the surgery. Secondary outcomes were the change in BCVA and CRT.

### Statistical analysis

Statistical Package for the Social Sciences (SPSS) software (version 21.0) were used for statistical analyses. Visual acuity was converted to the logarithm of the minimum angle of resolution (LogMAR) for statistical analysis. The data was analyzed in terms of normal distribution with Shapiro–Wilk test. As the distribution of the data was normal, visual acuity and the CRT values between baseline and the other time points were assessed with repeated measures test. Categorical variables were compared using Chi-square test. A *p* value < 0.05 was considered statistically significant.

## Results

Eleven eyes of 11 patients were included in the study. The mean age was 65.0 ± 5.5 years (range 61–80 years). Eight patients (72.7%) were female and 3 (27.3%) were male. The mean postoperative follow-up period was 14.8 ± 2.6 months (range 12–20 months). Six eyes (54.4%) were phakic and 5 (45.5%) were pseudophakic preoperatively. Two of the six phakic eyes (33.3%) underwent cataract surgery with phacoemulsification at the time of the vitreoretinal surgery. The general characteristics of the patients were listed in Table 1.

**Table 1:** General characteristics of the whole group

Patient no.	Age	Gender	Follow-op, months	Lens status	Cataract surgery	Preop BCVA, LogMAR	Month 12 BCVA, LogMAR	Preop CRT, µ	Month 12 CRT, µ	Peeled membrane	Tamponade	Postop foveal contour	Complication
1	64	Female	18	Phakic	–	1.00	0.52	540	324	ERM + ILM	SIVI	Incomplete	Cataract
2	68	Male	14	Phakic	+	0.52	0.70	381	388	ERM + ILM	HAVA	Absent	–
3	61	Female	13	Phakic	+	0.52	0.15	421	336	ERM	SIVI	Incomplete	–
4	61	Female	14	Phakic	–	0.70	0.52	556	350	ERM	SIVI	Incomplete	Cataract
5	61	Female	12	Phakic	–	0.70	0.70	268	264	ERM + ILM	SIVI	Complete	–
6	65	Female	18	Pseud	–	0.52	0.50	554	170	ERM + ILM	C3F8	Complete	RD
7	80	Male	20	Pseud	–	0.70	0.60	333	339	ERM + ILM	C3F8	Complete	–
8	61	Female	14	Pseud	–	0.52	0.40	581	347	ERM + ILM	SIVI	Incomplete	–
9	63	Female	14	Pseud	–	0.52	0.52	590	181	ERM + ILM	HAVA	Complete	–
10	65	Female	14	Phakic	–	0.52	0.30	660	333	ERM + ILM	SF6	Incomplete	Cataract
11	67	Male	12	Pseud	–	0.40	0.50	358	291	ERM	HAVA	Complete	–

no, number; BCVA; preop, peroperative; best corrected visual acuity; CRT, central retinal thickness; postop, postoperative; ERM, epiretinal mebrane; ILM, internal limiting membrane

The foveal contour was fully restored in 5 eyes (45.5%), partially restored in 5 eyes (45.5%), and was not restored in 1 eye (9.1%) at postoperative month 12 follow-up visit (Fig. 2).

**Fig. 4:**
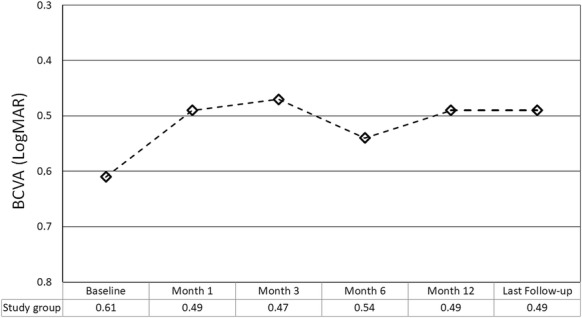
The change in best corrected visual acuity throughout the follow-up period (BCVA, best corrected visual acuity)

The mean preoperative BCVA was 0.61 ± 0.16 LogMAR (range 0.4–1.0 LogMAR). The mean BCVA at postoperative months 1, 3, 6, 12 and the last follow-up was 0.49 ± 0.12 (range 0.22–0.7), 0.47 ± 0.12 (range 0.22–0.7), 0.54 ± 0.22 (range 0.1–1.0), 0.49 ± 0.16 (range 0.15–0.7), and 0.49 ± 0.17 LogMAR (range 0.15–0.7), respectively (*p* < 0.0001 for all time points versus preoperative BCVA) (Fig. 4). Two eyes (18.1%) gained ≥ 3 lines of vision, the remaining 9 eyes (81.9%) remained stable (gained < 3 lines, did not change, or lost < 3 lines).

**Fig. 5:**
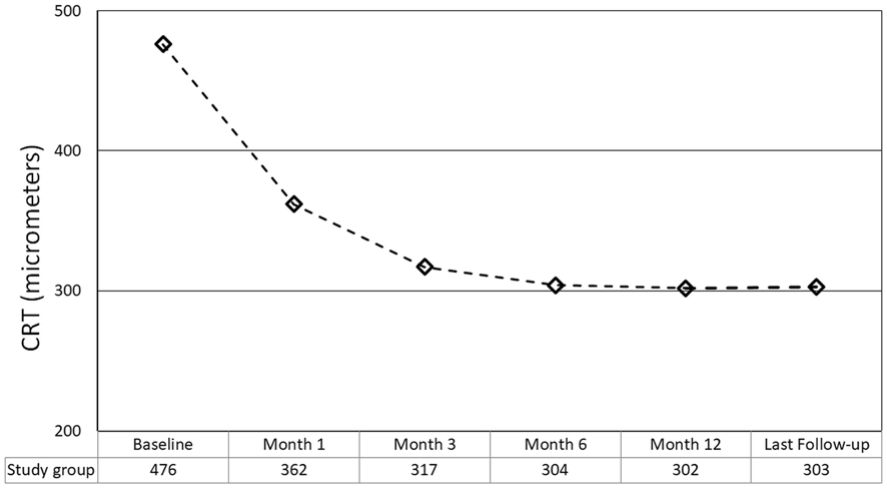
The change in central retinal thickness throughout the follow-up period (CRT, central retinal thickness)

The mean preoperative CRT was 476 ± 128 micrometers (range 268–660). The mean CRT at postoperative months 1, 3, 6, 12 and the last follow-up was 362 ± 44 (range 274–428), 317 ± 80 (range 120–385), 304 ± 79 (range 140–372), 302 ± 70 (range 170–388), and 303 ± 61 micrometers (range 180–370), respectively (*p* < 0.0001 for all time points versus preoperative BCVA) (Fig. 5).

Solely ERM was peeled in 3 eyes (27.3%), and both ERM and ILM were peeled in 8 eyes (72.7%). None of the eyes showed ERM recurrence during the follow-up period. The mean BCVA was 0.54 ± 0.15 LogMAR (range 0.4–0.7) and 0.64 ± 0.16 LogMAR (range 0.52–1.0) in patients in whom only ERM and both ERM and ILM were peeled, respectively (*p* = 0.3). The mean visual acuity was increased by 1.5 and 1.1 lines in only ERM and both ERM and ILM peeled eyes, respectively (*p* = 0.8). No tamponade was used in 5 (45.5%), air was used in 3 (27.3%), sulphur hexafluoride was used in 1 (9.1%), and perfluoropropane was used in 2 eyes (18.2%).

Three of the 4 remaining phakic eyes (75%) required cataract surgery during the follow-up period. No significant peroperative complications occurred during the surgeries. Only one eye had a previously treated peripheral retinal tear, and at postoperative month 2 this eye progressed to retinal detachment. This eye was operated again and had an attached retina at the last follow-up visit.

## Discussion

We reviewed the outcomes of vitrectomy in 11 patients with ERM associated with foveal herniation. Foveal contour was completely restored in nearly half of the patients and the visual acuity increased significantly in two patients at month 12. Also, the mean CRT decreased significantly at the postoperative period throughout the first year. The visual and anatomical outcomes of this study were similar to previous studies in terms of vitrectomy outcome in ERM patients [4–7, 11] We documented an increase of 1.2 LogMAR lines in visual acuity at month 12 in this study. Kinoshita et al. [7] evaluated the time course of changes in metamorphopsia, visual acuity and OCT parameters after ERM surgery in their study. The visual acuity was found to be increased from 0.38 LogMAR to 0.09 LogMAR at month 12 and 67.3% eyes showed ≥ 2 lines of visual increase. In a previous study by our group, we evaluated the outcomes of subtotal vitrectomy in patients with ERM and macular hole [11]. After a follow-up period of 12 months the BCVA increased from 0.65 LogMAR to 0.56 LogMAR. The change in BCVA was evaluated in a meta-analysis by Azuma et al. [5]. The change in mean BCVA ranged from − 0.05 LogMAR lines up to + 0.46 LogMAR lines in ERM + ILM peeled patients and between − 0.13 and + 0.42 LogMAR lines in only ERM peeled eyes. Internal limiting membrane peeling is an important adjuvant to ERM peeling in patients with ERM and has been to show to decrease the recurrence rate [5]. Azuma et al. [5] also evaluated the recurrence rate of ERMs in a total of 1201 eyes in their valuable meta-analysis. The rate of ERM recurrence was reported to be 2.0% (13/645 eyes) in ERM + ILM peeling group and 11.0% (66/600 eyes) in only ERM peeling group and the change was found to be statistically significant between the two groups. We used a standard technique of observing the integrity of ILM after the peeling of ERM. The integrity and continuity of ILM was checked with the help of brilliant blue G in all of our cases and we peeled the ILM only in the event of disruption or suspicion of disruption. In this study most of the eyes showed disruption of ILM after the peeling of ERM, and we peeled the ILM in 8 of 11 included eyes. None of our included patients showed recurrence of ERM during the follow-up period. This may be because of small sample size and also secondary to the high rate of ILM peeled eyes. We also evaluated the visual change at month 12 between the subgroups of patients in whom only ERM (1.6 lines) was peeled and both ERM and ILM (1.1 lines) were peeled, and did not find statistically significant difference between the subgroups.

Several types of epiretinal membranes were defined in previous studies [3, 4, 7–9, 12, 13]. Hwang et al. [8] were the first authors to define the later so called "foveal herniation" subtype as "outer retinal inward projection and inner retinal thickening. In that study, they mainly divided the ERMs into two main groups as fovea attaching (1) and fovea sparing groups (2) and the former one had 3 groups while the latter had 2. The foveal herniation group was defined as subtype 1B. They evaluated the macular function via multifocal electroretinography (mfERG) and reported that the eyes in group 2 had better foveal function than the eyes in group 1 in regards to mfERG outcomes. The study demonstrated further data concerning inter-group changes and correlation with the fellow eyes of the patients; however, this was a cross-sectional study and did not give longitudinal data about these patients. Kinoshita et al. [7] divided the ERMs into 4 groups as diffuse, cystoid macular edema, pseudolamellar hole, and vitreomacular traction (VMT) subtypes. The subtype of the ERM was evaluated as a predictive factor for the surgical outcome in the study, but there was not any further data and whether the included eyes showed a lesion like foveal herniation or not were not reported. They concluded that the VMT group showed better visual outcomes and also higher recurrence than the other groups. Although several authors also made different classifications according to morphology of ERMs on OCT, Ozdemir and Karacorlu were the first authors who used the term “ERM with foveal herniation” [10]. They reported a 65-year-old woman who had an ERM with foveal herniation in the left eye. The authors described the OCT appearance as the herniation of the superficial layers of the retina towards the vitreous through an opening in the center of the ERM.

Although it is a well-known fact that the formation of ERMs are related with PVD [14–16], data concerning the possible mechanisms of the formation of a foveal herniation and surgical outcomes are scarce in the literature. At the first stages of PVD, vitreous is found to be detached from the fovea, but attached to elsewhere around it [17, 18]. Perhaps, while posterior vitreous detaches from the retinal surface, the cortical vitreous might completely detach from the foveola but remain attached firmly around the fovea during these early stages. This may lead to the vitreous remnants on the retinal surface during the detachment of the posterior vitreous from the perifoveal area, a phenomenon previously described as vitreoschisis. If these remnants progress to ERM and contractile cells get involved into this process, this new ERM may create a centrifugal contraction and cause inner retinal layers to protrude from the central operculum to form a foveal herniation. Although this is-at least for now-a little bit hypothetical, it may be one of the possible mechanisms underlying the formation of foveal herniation.

The main limitations of this study were the small study population and its retrospective design. However, foveal herniation is a special and rare subtype of ERM. To the best of our knowledge, we evaluated for the first time the surgical outcomes of foveal herniation in 11 patients during a follow-up time of 12 months.

As a conclusion, the surgical outcomes were satisfactory and similar to the previous ERM series. Nearly half of the included eyes showed a fully formed foveal contour at month 12. Visual and anatomical outcomes were also acceptable and similar to the previous studies. Further studies with a longer follow-up period are required to evaluate the clinical significance of this specific subtype of ERM. Similarly, the true incidence of foveal herniation and its surgical outcomes are yet to be determined in larger series.

## Abbreviations

ERM: epiretinal membrane
BCVA: best corrected visual acuity
CRT: central retinal thickness
OCT: optical coherence tomography
PVD: posterior vitreous detachment
ILM: internal limiting membrane
LogMAR: logarithm of the minimum angle of resolution
VMT: vitreomacular traction
